# Deregulation of ribosomal proteins in human cancers

**DOI:** 10.1042/BSR20211577

**Published:** 2021-12-17

**Authors:** Wendy El Khoury, Zeina Nasr

**Affiliations:** Department of Biology, Faculty of Arts and Sciences, University of Balamand, Lebanon

**Keywords:** apoptosis, Cancer, cell survival, metastasis, ribosomal proteins, Ribosomes

## Abstract

The ribosome, the site for protein synthesis, is composed of ribosomal RNAs (rRNAs) and ribosomal proteins (RPs). The latter have been shown to have many ribosomal and extraribosomal functions. RPs are implicated in a variety of pathological processes, especially tumorigenesis and cell transformation. In this review, we will focus on the recent advances that shed light on the effects of RPs deregulation in different types of cancer and their roles in regulating the tumor cell fate.

## Introduction

Protein synthesis is a highly regulated and coordinated process involving the action of ribosomes and a set of translation factors. Ribosome biogenesis occurs in the nucleolus and requires the action of 80 ribosomal proteins (RPs), 4 ribosomal RNAs (rRNAs), other associated proteins and small nucleolar RNAs (snoRNAs) [1,2]. Ribosome biogenesis is a highly ordered process that requires a substantial amount of energy [3]. Deregulation of RPs can affect ribosomal biogenesis which can impair cell survival, growth and proliferation. Studies have shown that RPs have extraribosomal functions including roles in DNA repair, replication, proliferation, apoptosis and chemoresistance [4]. Indeed, mutations in RPs in animal models induce a wide variety of phenotypes ranging from decrease in body size, defective organs to embryonic lethality suggesting a role of RPs beyond the ribosome structure [4,5]. In mammalian cells, RPs deficiency leads to ribosomal stress; the deficiency in one RP leads to depletion of other RPs with deleterious effects on cell survival. Mutations in RPs lead to poor assembly into the ribosomal subunit and increased cancer risk [4,6]. In fact, several RPs are found overexpressed in various human tumors, including prostate cancer (PCa), colon adenocarcinomas, liver, pancreatic, gastric, lung and breast cancer among others [6,7]. RPs’ patterns of expression in various human tumors are highly specific to tumor types and tissues implicating them as biomarkers for various cancers, while others are involved in chemoresistance [7,8].

In addition, RPs have been linked to p53 [9], a tumor suppressor protein that is involved in the surveillance mechanism that keeps the integrity of the ribosomes and the cell. p53 is involved in the cell stress response, including ribosomal stress, usually inducing apoptosis, cell cycle arrest or senescence. Upon ribosomal stress, several RPs interact with MDM-2, the suppressor of p53, thus stabilizing p53 while deficiency in individual RPs can prompt p53 activation [10].

In normal conditions, p53 expression is low due to its interaction with MDM-2 that causes its ubiquitination and its degradation by the proteasome. However, upon ribosomal stress, induced by radiations, genotoxic agents or oxidizing chemicals, the release of many RPs from the ribosomes into the nucleoplasm may happen leading to the impairment of ribosome biogenesis and to the association of those RPs with MDM-2, suppressing the degradation of p53. In fact, genome-wide analyses across human cancers have tightly linked p53 mutations with RPs deletions which increase the vulnerability to human cancers, especially mutations in RPL5 and RPL11 [11,12].

In addition to the activation of the p53 pathway, some RPs are also shown to be involved in other mechanisms by modulating oncoproteins, or interacting with other signaling pathways such as mTOR, NFκB, wnt/β-catenin and let-7a, among others [13]. It is well known that the down-regulation of the mTOR pathway inhibits the translation of several RPs, while genome-wide analysis in pancreatic cancer cells have shown that RPs are among the top differentially translated genes upon treatment with mTOR inhibitor [14,15].

In the present review, we elucidate the effect of several deregulated RPs on different human cancer types as well as the mechanisms implicated in this process, in order to shed light on the importance of RPs as biomarkers or targets to treat tumor cells.

## RPs in colon cancer

Accumulating data have shown that many RPs are overexpressed in colorectal cancer (CRC) [16]. Analyzing the gene-expression profiles for almost 50 genes of 20 CRC tumors have shown that RPS3, S3A, S4X, S27a, L3, L6, L9, S2 are all overexpressed in colon cancer [17]. Kitahara et al*.* have demonstrated that RPL8, L18, L18a, L29, L6, L3, S19, L7 are all up-regulated in eight CRC tissues compared with normal tissues [18]. RPL35 and RPS23 are found to be overexpressed in both early- and late-stage of CRC [19]. Luo et al. have reported that the expression of RPS2 and RPS12 are increased in colon cancer tissues [20]. RPS15a, RPL18, RPL19, RPL31, RPS19, RPS27, RPL7, RPS11 and RPL15 are confirmed to be overexpressed in human colon cancer tissues compared with normal tissues in several studies [21–28].

Among these RPs, RPS3 has been shown to have high levels of expression in adenocarcinoma tissues compared with normal ones [29]. In one of these tumors, a high expression of RPS6, S8, S12, L5 and P0 is also observed which suggests that RPs could be regulated by mutual coordination. Moreover, Alam et al*.* have shown that RPS3 modulates the levels of p53 and lactate dehydrogenase (LDH) which affects the growth of colon cancer. The knockdown (KD) of RPS3 by small interfering RNA (siRNA) significantly impedes cell proliferation, invasion and migration while increasing apoptosis rates in colon cancer Caco-2 cells with no effect on normal colon mucosa NCM-460 cells. This has correlated with an increase in p53 expression and decrease in LDH levels exclusively in Caco-2 cells. These results demonstrate that RPS3 is involved in colon cancer growth and progression with minimal effect on normal cells which might suggest it as a potential therapeutic target and prognostic marker for colon cancer [30].

Similarly, the knockdown of RPS9, RPS15a and RPS27 inhibit human colon cancer cell growth and proliferation [21,25,31]. Targeting either RPS9 or RPS15a induces cell cycle arrest at the G_2_-M phase by down-regulating cyclin-dependent kinase 1 (CDK1) which suggest that these RPs could interact with the p53 pathway [21,31]. On the other hand, RPS27 KD is linked to inhibition of colon tumor progression by impeding the JNK/c-Jun signaling pathway and promoting leptin circulation, especially in obese CRC patients *in vivo* [25]. In fact, RPS27 up-regulation is associated with bad prognosis in obese leptin-induced CRC patients [25]. Interestingly, RPS27A, a family member of RPS27 similar to c-jun and c-fos, is shown to be also overexpressed in human colonic cancer [32]. In addition, RPS27-like (RPS27L) down-regulation is associated with poor prognosis in feces and tumor tissues of intermediate-stage CRC patients, while its overexpression and high p53 level promote better prognosis by enhancing the DNA repair capacity in LoVo cells [33]. This shows that intermediate-stage CRC patients, with consistent expression of p53 and RPS27L, have a better prognosis than patients whose level of p53 is not able to activate RPS27L.

In addition, Huang et al. have shown that RPL19 has been detected in the feces of late-stage CRC patients and correlate with poor prognosis when co-expressed with carcinoembryonic antigen (CEA). RT-PCR has revealed that RPL19 mRNA is up-regulated in late-stage colon cancer cell lines LOVO, Caco-2, HCT 116 and HT29, which indicates that RPL19 could be a prognostic marker in CRC [22].

On the other hand, the knockdown of RPL9 by siRNA has been linked to apoptosis in colon cancer cells *in vitro* and *in vivo*. RPL9 KD inactivates Id-1/nuclear factor-κB (NFкB) signaling pathway in HT29 and HCT116 colon cancer cells when compared with the control by phosphorylating IкB, which is known to prevent the translocation of NFкB into the nucleus and the activation of genes that promote cell survival. These results suggest that RPL9 could be a critical target to treat CRC [34].

RPL29 is up-regulated in colon cancer cells which correlates with the inhibition of apoptosis and promotion of the survival of the cancerous cells. A repression of RPL29 has been associated with cell differentiation in colon cancer cells and has shown that the expression of RPL29 is controlled by β-catenin/Tcf-4 pathway, which is an important pathway that controls the switch between cellular proliferation and differentiation in normal and malignant intestinal epithelial cells [35,36].

Similarly, the mRNA of RPS19 has been shown to be overexpressed in primary colon carcinoma tissues and present in both well and poorly differentiated cell lines [24]. A high level of RPS19 and laminin-binding protein (LBP) mRNAs, combined with low level of HLA-I, are linked to high malignancy of colon carcinoma. However, Chien et al. have demonstrated that RPS19 has a low expression in the feces of CRC patients with bad prognosis [37]. In fact, the mechanism through which RPS19 affects colon cancer is possibly through Bax/p53 pathway as the latter is known to be a negative prognostic factor in colon cancer when it is down-regulated [38].

On the other hand, some RPs are down-regulated in colon cancer. RPS5, in addition to four other genes (*BRI3*, *CHD2*, *MGC23401* and *ZNF148*), are shown to be down-regulated in colon cancer and associated with tumor progression [39]. RPS7 is also down-regulated in colon cancer and its overexpression is correlated with good prognosis as it suppresses hypoxia-inducible transcription factor-1α (HIF-1α), metabolic promoting proteins glucose transporter 4 (GLUT4) and lactate dehydrogenase B (LDHB) both *in vitro* and *in vivo* [40].

Russo et al. have demonstrated that RPL3 is down-regulated in colon cancer and inversely related to Bcl-2/Bax ratio. The activation and overexpression of RPL3 by the chemotherapeutic drug 5-fluorouracil (5-FU) induces cell apoptosis and regresses cell proliferation in colon cancer, which suggest that the combination of RPL3 and 5-FU could be a novel therapy in colon cancer [41]. This is supported by the fact that the absence of uL3, the cross-domain name of RPL3, can induce chemoresistance by increasing autophagic flux in colon cancer cells [42].

Kasai et al*.* have shown that ten RPs (Sa, S8, S12, S18, S24, L13a, L18, L28, L32, and L35a) are highly down-regulated in colon cancer cells compared with normal mucosa as confirmed by immunohistochemistry (IHC) analysis using an antibody panel. These RPs are highly expressed in the normal tissues, especially in the mature region of the mucosal epithelia compared with immature epithelial cells. Some contradicting data suggest that some of these RPs, such as RPS24 and RPL28, are overexpressed in CRC and their inhibition could lead to a better prognosis [43,44]. In fact, RPS24 KD by small hairpin shRNA in the human colon cancer HCT116 and HT-29 cell lines leads to an inhibition in proliferation and colony formation as well as induction of cell cycle arrest in S phase and decrease in G_2_-M phase [43]. Similarly, RPL28 KD decreases HCT116 and HT-29 cell proliferation *in vitro* [44] while its expression is higher in colorectal tissues compared with normal counterparts and also correlates with decreased survival rates in metastatic cases. These controversial findings need more analysis to pinpoint the exact role of these RPs in CRC by determining in which pathways they could be exerting their effects.

Genomic and functional studies have shown that mutations in the *RPS20* gene predispose patients to familial colorectal cancer type X (FCCX) which is a type of hereditary non-polyposis colorectal carcinoma. These mutations are linked to a deregulation in the maturation of pre-rRNA in these patients [45].

All these data indicate that many RPs could work as efficient prognostic markers in colon cancer and could be targeted for potential therapy. Several pathways seem to be involved with p53 being the major protein regulated by these RPs in different settings.

## RPs in prostate cancer

Many RPs are also shown to be differentially expressed in PCa. RPS19, RPS21 and RPS24 are found to be up-regulated in human cancerous prostate tissues, gathered from 82 patients, when compared with normal ones suggesting that these RPs could serve as biomarkers for PCa [46]. In addition, RPL21L and RPS21 are overexpressed in PCa with a higher expression in high Gleason grade compared with low Gleason grade cases. *In vitro* studies have revealed that these RPs promote cell proliferation and metastasis while inhibiting apoptosis in PCa cell lines [47]. This is also confirmed by Fan et al*.* who reported that many key genes and signaling pathways are implicated in PCa including RPS21 and RPL22L that are significantly up-regulated in PCa, suggesting that they could be used as potential prognostic markers [48].

Moreover, RPL31 is shown to be overexpressed in PCa tissues by affecting the levels of the tumor suppressor p53 and its targets, the cell-cycle negative regulator p21 and the E3 ubiquitin ligase targeting p53, MDM2. Upon the suppression of RPL31 using a lentiviral shRNA library in PCa cells LNCaP and BicR, an enhancement of the levels of p53, p21 and MDM2 has been demonstrated by Western Blot, which is associated with a decrease in cell growth. Transfection with p53 siRNA recovers this suppression of cell growth upon RPL31 knockdown, which further proves that this RP is implicated in PCa growth and development in a p53-dependent manner [49].

Another example of a highly expressed RP in PCa is RPS2. This protein promotes tumorigenesis in all PCa tissues when compared with their normal counterparts. It is proposed that RPS2 blocks the expression of let-7a which is known to inhibit Ras and Myc activation by binding to pre-let-7a. Therefore, the inhibition of let-7a promotes the activation of Ras, and possibly Myc or p53, which may be linked to the tumor growth in PCa [50].

RPS6KB1 is well demonstrated to be up-regulated in PCa and associated with tumor growth and stage. RPS6KB1 expression decreases in the presence of Nexrutine (Nx), a natural compound that inhibits PCa tumor growth in combination with radiotherapy. The KD of RPS6KB1 can increase the sensitivity of PCa cells to radiotherapy and inhibit their survival *in vitro* and *in vivo* [51]. Similarly, palmatine, a subfraction of Nx, is able to inhibit PCa growth and invasion by concurrently targeting RPS6 and NFκB/FLIP pathway [52]. The expression of phosphorylated mammalian target of rapamycin complex 1 (p-mTORC1), the upstream regulator of RPS6 is also increased in PCa tissues and correlates with a high expression of p-RPS6 and p70 RPS6 kinase 1 (p70^S6K1^) as shown by IHC analysis [53]. These data suggest that RPS6 could play an important role in PCa growth and could be targeted for efficient therapy.

RPS7 is reported to be overexpressed in PCa and closely associated with tumor growth and invasion via epithelial–mesenchymal transition (EMT). In fact, knockdown of RPS7 could participate in the up-regulation of the epithelial protein marker E-cadherin and the down-regulation of the mesenchymal protein markers, such as N-cadherin and Snail and subsequently attenuating prostate tumor growth [54]. Moreover, RPS7 overexpression in PCa is associated with poor prognosis. Interestingly, an inhibition of RPS7 and PIM1, a member of PIM family and an important player in the control of cell growth, apoptosis and cell cycle progression, has been linked to the stabilization of the oncogene c-Myc and a reduction in tumor growth *in vitro* and *in vivo* [55].

According to Bee et al*.*, the overexpression of RPL19 in PCa could lead to tumor growth and is inversely correlated with the patients’ survival rate. Interestingly, knockdown of RPL19 by siRNA avoids prostatic malignancy *in vitro* and *in vivo* in PC-3M PCa cell lines suggesting that RPL19 could be used as a prognostic marker [56,57].

Slot-blot hybridization studies have demonstrated that RPL4, L5, L7a, L23a, L30, L37, S14 and S18 mRNA levels are higher in at least one of the three types of PCa cell lines LNCaP, DU-145 and PC-3 and the overexpression of RPL7a and RPL37 is confirmed in PCa tissues [58]. Using cDNA microarray analysis, it has been reported that among the differentially expressed genes in PCa cell lines LNCaP, RPL10, RPL32 and RPS16 are shown to be overexpressed in these cell lines [59].

All these data indicate that a wide variety of RPs are up-regulated in PCa and could be used as potential biomarkers or targeted for effective treatment.

## RPs in breast cancer

RPs are known to have many extraribosomal functions involved in the regulation of breast cancer growth and development. Some RPs have been shown to be overexpressed in breast cancer in different settings. RPS3, which is known to interact with NFκB, is overexpressed in breast cancer by up-regulating the X-linked inhibitor of apoptosis (XIAP) [60]. The knockdown of RPS3 in human breast cancer cells induces a decrease in proliferation and increase in apoptosis which correlates with a reduction in the levels of the XIAP protein, with no effect on NFκB activity. These results suggest that RPS3 is involved in human breast cancer in an NFκB-independent pathway [61].

In breast tumor tissues, a higher expression of RPS15A is detected and correlates with larger tumor size and higher TNM stage [62]. In MDA-MB-231 cell lines, the knockdown of RPS15A suppresses breast cancer proliferation and induces apoptosis by increasing the caspase 3/7 activity, and suppressing the phosphorylated levels of ERK1/2, Bad and Chk1. Indeed, the expression of RPS15A leads to an inhibition of apoptosis by up-regulating phosphorylated ERK1/2, Bad and Chk1 [62]. The knockdown of RPS15A by shRNA in breast cancer cell line ZR-75-30 and BT474 mediates apoptosis via the activation of caspase-3 and PARP cleavage, the up-regulation of Bad and BAX and the down-regulation of Bcl-2 confirming the importance of RPS15 in the modulation of breast cancer development [63].

RPS19 and RPL39 are also demonstrated to be overexpressed in breast cancer. RPS19 induces the production of immunosuppressive cytokines and promotes tumor growth which can be impaired by the blockage of RPS19 [64] while the knockdown of RPL39 in triple-negative breast cancer (TNBC) xenografts reduces significantly primary tumor growth, as well as metastasis [65].

In addition, knockdown of RPS6 is shown to be linked to a reduction in breast cancer cell proliferation and viability [66]. RPS6 expression decreases in cells treated with a combination of epithelial growth factor receptor (EGFR) and mesenchymal–epithelial transition (MET) inhibitors *in vitro* which correlate with a reduction in cell proliferation and survival as well as induction of cell cycle arrest [66].

RPL24 level is shown to be higher in breast cancer tissues compared with their normal counterparts. The knockdown of RPL24 decreases cell viability by reducing the level of the cell cycle regulator cyclin D1, the anti-apoptotic protein survivin and NBS1 which is involved in the regulation of DNA damage response [67].

Hong, Kim, and Kim have shown that RPL19 is also up-regulated in breast cancer [68]. Overexpression of RPL19 induces pre-activation of the unfolded protein response (UPR), including phosphorylation of pERK-like ER kinase (PERK), phosphorylation of eukaryotic translation initiation factor 2 α (eIF2a), and activation of p38 MAPK-associated stress signaling which enhanced cell death in MCF7 breast cancer cells [68].

An *in vivo* genome-wide CRISPR activation screen has identified the overexpression of RPL15 in breast cancer patient-derived circulating tumor cells in mice. The high levels of RL15 are linked to an increase in metastatic growth and enhancement of translation of other RPs and cell cycle regulators suggesting the role of RPL15 in breast cancer metastasis [69].

RPL32 is also found to be up-regulated in human breast cancer tissues and cells. The knockdown of RPL32 by a lentivirus-delivered siRNA has a negative effect on the migration and invasion of breast cancer cells *in vitro* and *in vivo*. RPL32 KD also decreases the expression levels of matrix metalloproteinase (MMP)-2 and MMP-9, which are important in tumor cell invasion and metastasis. These results indicate that RPL32 could be a novel target to treat patients with advanced breast cancer [70].

On the other hand, the low expression levels of RPS9, RPS14, RPS27, RPL11 and RPL14 are related to a poor overall survival in breast cancer patients, especially in TNBC [71]. These five genes could play a role in the detection and treatment of this type of cancer. RPL5 down-regulation in breast cancer is also associated with a poor prognosis. Knockdown of RPL5 in breast cancer cell lines promotes G_2_/M cell cycle progression and enhances tumor growth in xenograft mouse model which confirms the potential suppressive role of RPL5 in breast cancer [72].

Other RPs are found to be down-regulated in breast cancer including RPS27L. The knockdown of RPS27L induces autophagy in breast cancer MB231 and SK-BR3 cells by shortening the protein half-life of β-TrCP which is involved in the degradation of DEPTOR, the natural inhibitor of mTOR. The accumulation of DEPTOR inactivates mTORC1 and induces autophagy. The blockage of autophagy induced by RPS27L leads to the regression of breast cancer cell growth by triggering apoptosis [73]. These results point to the involvement of the β-TrCP-DEPTOR-mTOR axis in the progression of breast cancer in patients with low level of RPS27L.

## RPs in liver cancer

RPs expression is also modulated in liver cancer. The most common type of this cancer is hepatocellular carcinoma (HCC) where many RPs are known to be differently regulated. Among these, RPL12, L23a, L27, L3, SA and S2 are shown to be up-regulated in HCC. The latter is known to induce tumor proliferation *in vitro* and *in vivo* [74–76]. In the case of RPSA, the overexpression of the microRNA-587 (miR-587) inhibits RPSA expression and reduces cell proliferation and invasion in HCC cell line SMMC-7721 [76]. Similarly, RPL39L overexpression is correlated with increased tumor invasion and poor prognosis in HCC cells [77].

RPL36 is found to be up-regulated in 75% of 60 HCC patients especially in early stages of carcinogenesis suggesting its potential role as a biomarker in liver cancer [78]. On the other hand, RPS8 is found to be up-regulated in alcohol-associated HCC and not in non-alcoholic-associated HCC which makes RPS8 a biomarker and a novel therapeutic target for HCC linked to alcohol [79].

Moreover, RPS6K, which phosphorylates RPS6, is up-regulated in HCC patients when compared with normal ones, and this up-regulation is linked to the activation of Akt-mTOR-p70S6K signaling pathway that promotes neoangiogenesis in HCC [80,81]. In fact, the AKT/mTORC1/RPS6 pathway is involved in the regulation of lipogenesis, an oncogenic event in liver cancer *in vitro* and *in vivo* [82]. This axis is also dampened upon the knockdown of RPS4 X-linked (RPS4X) in HCC cell lines which correlate with a decrease in cell proliferation, migration and invasion and an increase in apoptosis [83]. These results indicate that the AKT/mTORC1/RPS6 axis is a potential marker in HCC but the precise function of the phosphorylation of RPS6 in this pathway needs further elucidation.

In HCC cells HepG2, the knockdown of RPS15A by shRNA causes cell cycle arrest at G_0_/G_1_ phase suggesting that RPS15A could be a potential therapeutic target in liver cancer [84]. Indeed, the overexpression of RPS15A in HCC is linked to an increase in fibroblast growth factor 18 (FGF18) expression following the stimulation of Wnt/β-catenin pathway which induces tumor angiogenesis [85].

It is also reported that RPS3 is up-regulated in HCC and can promote hepatocarcinogenesis *in vitro* and *in vivo* by stabilizing its target, the silent information regulator 1 (SIRT1) mRNA at the post-transcriptional level. The inhibition of RPS3/SIRT1 pathway by 5-formylfuran-2-yl methyl 4-hydroxy-2-methylenebutanoate (FMHM) represses HCC progression suggesting that RPS3/SIRT1 could serve as a potential therapeutic target in liver cancer [86].

RPL4 is reported to be up-regulated in HCC cells when compared with normal ones and associates with poor prognosis [87]. Yang et al. have shown that the level of RPL4 mRNA is significantly reduced in HCC cells upon the knockdown of the lncRNA small nucleolar RNA host gene 7 (*SNHG7*), which plays a pivotal role in HCC metastasis. The restoration of RPL4 level reverses the effect of SNHG7 on liver cancer cells. These results indicate that RPL4 targeting could play a potential role in HCC treatment [88].

In addition, a chronic infection with hepatitis B virus (HBV) could lead to HCC development especially through the factor hepatitis B virus X protein (HBx) which is detected in many cases of HBV-related HCC patients and can activate diverse signaling pathways related to tumorigenesis [89]. Several RPs such as the acidic ribosomal phosphoprotein P0, RPS20, L8, L27a, L21, L31, L35a, L37a, S24, S27a, S8, L37a, S3a, S15a and S5 are found to be up-regulated in HBV-associated HCC [90–92]. Among these RPs, RPS3a has been found to interact with HBx via its chaperoning activity and subsequently stimulate the activation of the oncogenic signaling pathway NFκB in HCC [93]. Similarly, RPS27A is found to be overexpressed in HBx-expressing HCC cell lines and in the liver of transgenic model of HCC. RPS27A inhibition induces significant reduction in cell proliferation and cell cycle arrest either at G_0_/G_1_ phase in HCC cell lines [94]. However, RPS27A is down-regulated in virus-induced HCC tissues compared with normal liver tissues and correlates with the overexpression of a cold shock domain family protein, Y box-binding protein (YB-1), that is known to be associated with several RPs and induce carcinogenesis [95]. The differential expression of RPS27A in different settings and the possibility for targeting it for HCC therapy needs to be further explored.

RPL5 and RPL11 are known to interact with MDM-2 and stabilize p53 by inhibiting its ubiquitination and degradation. This pathway seems to be highly involved in HCC progression. A germline copy number variation (CNV)-based genome-wide association study (GWAS) have identified the overexpression of small nucleolar RNA H/ACA box 18-like 5 (SNORA18L5) to be associated with a high risk of HBV-associated HCC [96]. SNORA18L5 expression is found to increase ribosome biogenesis causing RPL5 and RPL11 to stay in the nucleolus, which alter their localization and their interaction with MDM2 inducing p53 ubiquitination and degradation. This correlates with a decrease in p53-dependent cell cycle arrest and apoptosis in HCC cell lines [96]. Moreover, c-Myc inhibition leads to the up-regulation of RPL5 and L11 in HCC [97]. In fact, c-Myc is inhibited by a depletion of the midline 1 interacting protein 1 (MID1IP1), one of the glucose-responsive genes that is found to be overexpressed in HCC cells. However, an attenuation of RPL5 and RPL11 activates c-Myc in MID1IP1-depleted HCC cells. These data clearly suggest that RPL5 and L11 could serve as tumor suppressors in liver cancer.

## RPs in pancreatic cancer

RPs may also play a role in the development and progression of pancreatic cancer which is considered one of the most malignant types of tumors. It is reported that RPSA is highly expressed in the invasive human pancreatic cancer cell line PC-1.0 and is linked to Integrin α 6 (ITGA6), a protein involved in the regulation of cell–cell attachment [98]. ITGA6 promotes the activation of PI3K after regulating the phosphorylation level of AKT, while RPSA activates MAPK signaling pathway which consequently stimulates the invasion and the metastasis of pancreatic cancer cells. Knockdown of RPSA and ITGA-6 inhibits these pathways and leads to a reduction in pancreatic cancer cell line invasion and metastasis [98]. RPSA could also form a complex with the transient receptor potential melastatin-related 7 (TRPM7) that regulates pancreatic ductal cancer cell migration [99] suggesting that RPSA could work through different pathways and be a promising target in the treatment of this cancer.

Knockdown of RPL34 is also shown to have a negative effect on pancreatic cancer cells by inhibiting tumor proliferation, metastasis and promoting apoptosis. *In vivo* assays confirm that RPL34 affects pancreatic cancer cells through MAPK and p53 pathways which suggest that this RP could serve as a potential biomarker to detect and treat pancreatic cancer [100].

In addition, RPS15A is highly expressed in pancreatic cancer cell lines and is inversely correlated with the tumor suppressor miR-519d-3p expression. Inhibition of RPS15A by miR-519d-3p leads to the down-regulation of Wnt/β-catenin pathway in pancreatic cancer which proves the potential role of RPS15A as a target to treat pancreatic cancer [101].

RPs effect has been linked to KRAS, an oncogene mutated in 90% of pancreatic cancer. RPL26 and L29 are up-regulated upon knockdown of KRAS leading to cell proliferation of PANC-1 cell lines [102]. Inhibition of RPL26 and RPL29 in these cells is associated with reduced proliferation, increased apoptosis and blockage of cell cycle, which shed light on the importance of targeting these RPs as a potential therapy of pancreatic cancer. Muro et al*.* have discovered that the use of serum anti-RPL29 antibody retards pancreatic cancer cells proliferation *in vitro* suggesting that serum anti-RPL29 could be a biomarker in this cancer [103].

In addition, pancreatic acinar cells that express mutant KRAS or are treated with 7,12-dimethylbenz(a)anthracene (DMBA) show increase in RPS6 phosphorylation and expression. Phosphorylated P-RPS6 induces DNA damage triggered by the expression of the mutant KRAS and reduces p53-mediated tumor suppression which reveals that p-RPS6 is an important target in pancreatic cancer at initial stages [104].

RPL39 knockdown by siRNA in pancreatic cancer is also linked to cancer cell regression and apoptosis enhancement *in vivo* and *in vitro* [105]. RPL39 is overexpressed in the most aggressive pancreatic cancer cell lines PANC-1 and MIA PaCa-2. The effect of RPL39 on pancreatic cancer cell apoptosis seems to be dependent on caspase 8 activation suggesting that targeting RPL39 could be a potential treatment in pancreatic cancer [105].

RPL10 is another example of the deregulated RPs in pancreatic cancer. When pancreatic cell lines are treated with dimethylaminoparthenolide (DMAPT), a water-soluble agent that has anti-tumor activities, RPL10 expression is reduced. DMAPT can directly bind to RPL10 which consequently down-regulate the expression of p65 and IKKγ that are part of the NFκB signaling pathway inhibiting cell proliferation [106].

RPS8, RPL15 and RPL21 could also serve as biomarkers in pancreatic cancer. RPS8 overexpression is associated with short survival times in pancreatic cancer patients compared with the ones who express a lower level of RPS8 [107]. Yan et al*.* have reported that a low expression of RPL15 is related to a poor prognosis, as well as cell invasion and metastasis in pancreatic cancer patients. A high expression of this RP is inversely correlated with cell differentiation and metastatic stage classifications suggesting that RPL15 is an important indicator in this cancer [108]. In its turn, RPL21 KD by siRNA inhibits cell proliferation and DNA replication as well as induces G_1_ cell cycle arrest in pancreatic cancer cells PANC-1 and Bxpc-3, *in vitro* and *in vivo*. RPL21 KD promotes apoptosis in these two types of cells but not in normal ones by increasing caspase 8 activity, suggesting that targeting RPL21 could be an effective anti-cancer therapy [109].

## RPs in gastric cancer

Many RPs are found affected in gastric cancer (GC) including RPS13, RPL23, RPS27, RPL6, RPL13 and RPL15 [110–114]. Indeed, RPS13 and RPL6 are overexpressed in GC and can enhance colony formation *in vitro* as well as tumor formation *in vivo* and stimulate the G_1_/S transition in GC cells by inhibiting p27^kip1^ due to the up-regulation of cyclin E expression [115,116]. Similarly, RPS13 and RPL23 are overexpressed in GC and can promote multidrug resistance in GC cells by inhibiting drug-induced apoptosis. RPL23 may induce multidrug resistance by regulating glutathione S-transferase-mediated drug-detoxifying system [110].

RPS15A is also found to be up-regulated in GC. The knockdown of RPS15A by shRNA is linked to a reduction in cell proliferation, metastasis and to an arrest in the G_0_/G_1_ phase of the cell cycle [117]. RPS15A can activate the NFκB pathway through Akt/IKK-β signaling axis, thus promoting GC metastasis, suggesting that RPS15A could be a biomarker in GC [118].

In its turn, RPL15 is overexpressed in GC and associated with an increase in cell proliferation [114]. RPL15 can act as an interacting partner of p56, a gene encoding antiviral proteins activated by Type I interferons. Overexpression of p56 inhibits the growth of GC cells that overexpress RPL15 [119].

Some RPs are involved in the modulation of GC resistance to therapy. High levels of RPL11 in GC patients treated with 5-FU is associated with good prognosis. *In vitro*, it was demonstrated that RPL11 increases the sensitivity of GC cell lines to 5-FU by activating the p53 pathway suggesting the potential role of increased level of RPL11 in combination with 5-FU in the efficient treatment of GC [120]. In HER2-Amplified Gastric Cancer, RPS6 seems to be involved in resistance to therapy. Targeting RPS6 by siRNA or drug inhibitors induces tumor regression in resistant cells and models both *in vitro* and *in vivo* [121]. The PI3/AKT/mTOR/RPS6 pathway confers resistance in HER2-Amplified Gastric Cancer through the stimulation of the nuclear factor erythroid 2-related factor 2 (NRF2), a factor involved in chemo- and radioresistance in different tumors [121].

## RPs in lung cancer

RPs also play a critical role in lung cancer. For example, RPL9 is shown to be overexpressed in different types of lung cancer tissues with the highest expression in small cell lung carcinoma while RPS3A is highly expressed in squamous cell carcinoma indicating that these RPs could be considered as potential biomarkers in specific types of lung cancer [122,123]. Similarly, RPL34 is up-regulated in non-small cell lung carcinoma (NSCLC) tissues when compared with normal ones. Knockdown of RPL34 by a lentivirus-mediated shRNA in NSCLC cell line H1299 is correlated to a significant decrease in cancer cell proliferation and increase in apoptosis and S-phase arrest, suggesting that RPL34 may play an important role in the development of NSCLC [124].

Hyper-phosphorylated RPS6 (p-RPS6) is found to be associated with an unfavorable survival of NSCLC patients. The knockdown of RPS6, along with p-RPS6 alterations, induces cell cycle arrest at G_0_/G_1_ phase especially through the abnormal regulation of AKT2/mTOR/p70S6K signaling pathway suggesting that targeting RPS6 could be an effective therapy for NSCLC [125].

On the contrary, it has been reported that RPL22 is down-regulated in NSCLC suggesting that the low expression of RPL22 could play a potential role in the carcinogenesis of NSCLC [126].

*In vitro*, RPL19 is overexpressed in lung cancer H1224L cell line and positively correlates with interferon γ (IFN-γ) production. Inhibition of RPL19 by siRNA is linked to a regression in lung cancer progression and suppression in cyclinD1 and D3 syntheses [127]. Similarly, down-regulation of RPS15A by siRNA is found to be linked with a decrease in lung cancer A549 cells proliferation through G_0_/G_1_ cell cycle arrest suggesting that these RPs could function as potential biomarkers in lung cancer [128].

Some RPs have been correlated with apoptosis modulation in lung cancer. Yang et al*.* have demonstrated that phosphorylation of RPS3 and anti-apoptotic TRAF2 protein leads to radioresistance in NSCLC. Indeed, ionizing radiation (IR), in radioresistant NSCLC cells, leads to the phosphorylation of RPS3 and TRAF2 by casein kinase 2α (CK2α) and PKC, respectively, which promote the dissociation of RPS3–TRAF2 complex and the activation of NFκB. This activation is linked to a significant up-regulation of prosurvival genes, including *cIAP1*, *cIAP2* and survivin. However, this phenomenon is not detected in radiosensible NSCLC cells [129]. In addition, IR can induce a dissociation of the macrophage migration inhibitory factor (MIF) from RPS3, which can lead to an increase in pro-inflammatory cytokines secretion and a modulation in the expression of EMT marker proteins *in vitro* and *in vivo* [130].

RPS29 has been shown to induce apoptosis and improve the effect of anticancer drugs by down-regulating the expression of anti-apoptotic proteins Bcl-2, Bcl-X(L) and survivin, while up-regulating the expression of pro-apoptotic proteins p53 and Bax as confirmed by Western blot analysis in H520 cells. These modulations correlate with the release of cytochrome *c* from the mitochondria and the activation of initiator caspase-8 and 9 and effector caspase-3 suggesting the potential role of RPS29 as a biomarker in NSCLC [131].

5-FU chemotherapy treatment induces the production of RPL3 in Calu-6 cells that is found to have a role in negatively regulating the activation of NFκB through IκB-α up-regulation. Furthermore, RPL3 significantly enhances the apoptosis of Calu-6 cells which lead to the overexpression of the pro-apoptotic protein Bax and the inhibition of the anti-apoptotic protein Bcl-2 [132].

Other RPs can modulate the progression of lung cancer by affecting the DNA repair mechanisms. RPS27L has been shown to directly bind to two proteins involved in DNA interstrand cross-link repair, FANCD2 and FANCI, which protect them from degradation in lung cancer cells. The KD of RPS27L abrogates the level of FANCD2 and FANCI which sensitizes the cells to mitomycin C treatment reducing cell viability and colony formation [133].

These results indicate that the effects of RPs on lung cancer are dependent on the types of cancer and the different pathways through which these proteins are involved.

## RPs in other types of cancer

In addition to the major types of cancers mentioned above in which RPs can play a critical extraribsosomal role, these proteins have been also detected in other types of tumors. In here, we mention some examples including RPL34 that is up-regulated in esophageal cancer and its KD inhibits cancer cell proliferation, migration and invasion *in vitro* by down-regulating the protein expression level of p-PI3K and p-Akt [134]. RPS15A is overexpressed in renal cell carcinoma and related to tumor growth as its inhibition promotes apoptosis [135]. Moreover, mutations affecting RPL5 and RPL10 are present in 9.8% of pediatric T-cell acute lymphoblastic leukemia (T-ALLs) [136]. RPL10 mutation in T-ALL cells affects proliferation of these cells by increasing the reactive oxygen species (ROS) levels and BCL-2 expression highlighting the involvement of mutated RPs in tumorigenesis [137].

In nasopharyngeal cancer, it has been discovered that four RP genes *uS8 (S8), uS4 (S9), eS31 (S27a)* and *uL14 (L23)* are down-regulated in cancer cell lines suggesting that these RPs could serve as biomarkers in the carcinogenesis of nasopharyngeal cancer [138]. Expression of RPS7 is found to be linked with ovarian tumorigenesis and metastasis suppression. Silencing of RPS7 enhances ovarian cancer cell migration and invasion through PI3K/AKT and MAPK signal pathways [139]. RPS6 is found to be highly expressed in glioblastoma (GBM) tissues especially in the stem cell niche. RPS6 KD significantly dampens the stem cell-like features of GBM including a decrease in tumorsphere formation and the stem cell marker STAT3 expression indicating the importance of RPS6 in the maintenance of stem cell characteristics of GBM which are known to be the major reason for cancer relapse and resistance to therapy [140].

In cervical cancer, RPLP0 KD decreases cell viability and proliferation while increasing apoptosis level. These effects correlate with a modulation of cell cycle and apoptosis-associated proteins. In cells overexpressing the tumor suppressor gene phospholipase A and acyltransferase 4 (PLAAT4), RPLPO levels are decreased suggesting that the ratio of RPLP0 to PLAAT4 is an important regulator of cervical cancer growth and development [141].

## Conclusion and perspectives

**Figure 1:**
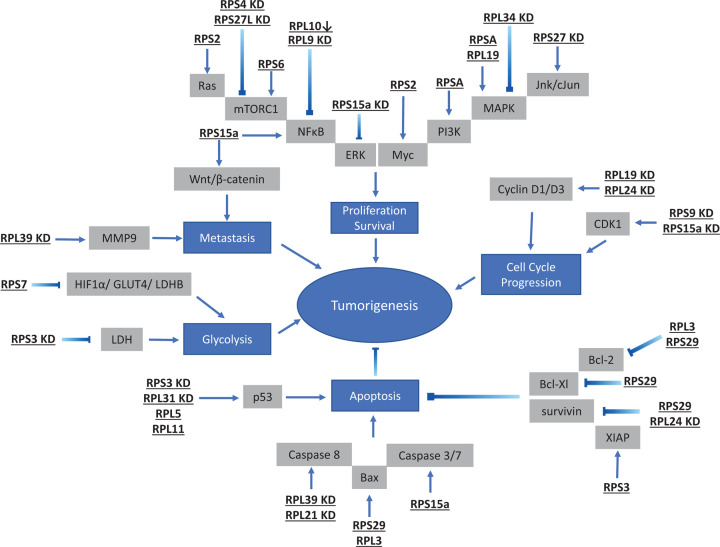
A representation of the most recent findings involving various RPs in the major known oncogenic pathways

The link between RPs and cancer is clearly established in this review. Table 1 summarizes the level of expression of the major RPs in several types of cancer with an emphasis on the target pathways involved. In conclusion, RPs seem to play crucial roles in different settings ranging from cancer onset and progression to metastasis. These proteins are involved in many pathways including those involved in cell survival, cell cycle progression, DNA repair and apoptosis (Figure 1). In the future, more work is needed to efficiently use RPs as biomarkers for either good or bad prognosis depending on the tumor type and stage. More importantly, it is crucial to start identifying drugs that can target these different proteins for efficient treatment of specific types of cancer.

**Table 1:** Expression of RPs in common human cancers with an emphasis on the targets involved in this deregulation

Type of cancer	Ribosomal proteins	Level of expression	Targets	Reference
**Colon cancer**	RPS3	Increased	p53 and LDH	[29,30]
	RPS9, RPS15a	Increased	CDK1	[21,31]
	RPS27	Increased	JNK/c-Jun	[25]
	RPL9	Increased	NFκB	[34]
	RPS2, S3A, S4X, S5, S6, S8, S11, S12, S19, S23, S24, S27a, L3, L5, L6, L7, L8, L15, L18, L18a, L19, L28, L29, L31, L35, and P0	Increased	**Not available**	
	RPS5	Decreased	HIF-1α, GLUT4, LDHB	[40]
	RPSa, S7, S8, S12, S18, S24, L3, L13a, L18, L28, L32, and L35a	Decreased	**Not available**	
**Prostate cancer**	RPL31	Increased	p53 and its targets: p21 and MDM2	[48]
	RPS2	Increased	let-7a	[50]
	RPS7	Increased	E-cadherin, N-cadherin and Snail; c-Myc	[54,55]
	RPS6, S6K, S14S16, S18, S19, S21, S24, L4, L5, L7a, L10, L19, L21L, L22L, L23a, L30, L32, and L37	Increased	**Not available**	
**Breast cancer**	RPS3	Increased	XIAP	[60]
	RPS15A	Increased	Caspase 3/7; ERK1/2, Bad and Chk1	[62]
	RPL24	Increased	Cyclin D1, survivin and NBS1	[67]
	RPL19	Increased	UPR, including PERK, eIF2a, and p38 MAPK-associated stress signaling pathway	[68]
	RPL32	Increased	MMP-2 and MMP-9	[70]
	RPS6, S19, L15, and L39	Increased	**Not available**	
	RPL5	Decreased	G_2_/M cell cycle	[72]
	RPS27L	Decreased	β-TrCP	[73]
	RPS9, S14, S27, S27L, L11 and L14	Decreased	**Not available**	
**Liver cancer**	RPS6K	Increased	Akt-mTOR-p70S6K signaling pathway	[80,81]
	RPS15A	Increased	Wnt/β-catenin pathway	[85]
	RPS3	Increased	SIRT1	[86]
	RPS3a	Increased	NFκB	[93]
	RPL5, L11	Increased	MDM2	[96]
	RPSA, S2, S4X, S5, S6, S20, S24, S27A, S8, L4, L5, L8, L11, L12, L21, L23a, L27, L27a, L30, L31, L35a, L36, L37a, L39L and P0	Increased	**Not available**	
	RPS27A	Decreased	YB-1	[95]
**Pancreatic cancer**	RPSA	Increased	MAPK; TRPM7	[98]
	RPL34	Increased	MAPK and p53	[100]
	RPS15A	Increased	Wnt/β-catenin pathway	[101]
	P-RPS6	Increased	p53	[104]
	RPL10	Increased	p65 and IKKγ	[106]
	RPL21	Increased	Caspase 8	[109]
	RPS6KA2, S8, L26, L29, L34 and L39	Increased	**Not available**	
	RPL15	Decreased	**Not available**	
**Gastric cancer**	RPS13, L6	Increased	p27^kip1^	[115,116]
	RPL23	Increased	Glutathione S-transferase-mediated drug-detoxifying system	[110]
	RPS15A	Increased	NFκB pathway through Akt/IKK-β signaling axis	[117]
	RPL15	Increased	p56	[119]
	RPS6	Increased	PI3/AKT/mTOR/RPS6 pathway	[121]
	RPS27, L13 and L23	Increased	**Not available**	
	RPL11	Decreased	p53 pathway	[120]
**Lung cancer**	RPS6	Increased	AKT2/mTOR/p70S6K	[125]
	RPL19	Increased	cyclinD1 and D3	[127]
	RPS27L	Increased	FANCD2 and FANCI	[133]
	RPS3, S3A, S15A, S27L, L9, L19 and L34	Increased	**Not available**	
	RPL3	Decreased	IκB-α	[132]
	RPS29	Decreased	Down-regulating Bcl-2, Bcl-X(L) and survivin; up-regulating p53 and Bax	[131]
	RPL22	Decreased	**Not available**	

## Abbreviations

CRC: colorectal cancer
DMAPT: dimethylaminoparthenolide
EMT: epithelial–mesenchymal transition
GBM: glioblastoma
GC: gastric cancer
HBV: hepatitis B virus
HBx: hepatitis B virus X protein
HCC: hepatocellular carcinoma
IHC: immunohistochemistry
IR: ionizing radiation
ITGA6: Integrin α 6
KD: knockdown
LDH: lactate dehydrogenase
MID1IP1: midline 1 interacting protein 1
NFκB: nuclear factor-κB
NSCLC: non-small cell lung carcinoma/cancer
Nx: Nexrutine
PCa: prostate cancer
RP: ribosomal protein
RPS27L: RPS27-like
RPSA: ribosomal protein A
rRNA: ribosomal RNA
siRNA: small interfering RNA
SNHG7: small nucleolar RNA host gene 7
SNORA18L5: small nucleolar RNA H/ACA box 18-like 5
TNBC: triple-negative breast cancer
TNM: tumor, nodes and metastasis
T-ALL: T-cell acute lymphoblastic leukemia
XIAP: X-linked inhibitor of apoptosis
5-FU: 5-fluorouracil
